# Edaravone Improves the Post-traumatic Brain Injury Dysfunction in Learning and Memory by Modulating Nrf2/ARE Signal Pathway

**DOI:** 10.6061/clinics/2021/e3131

**Published:** 2021-11-23

**Authors:** Xiushan Li, Jing Yu, Dongzhou Ma, Xuehui Weng

**Affiliations:** Department of Neurosurgery, Affiliated Hospital of Hebei University of Engineering, Handan, Hebei 056002, China.

**Keywords:** Edaravone, Traumatic Brain Injuries, Dysfunction in Learning and Memory, NSCs, Nrf2/ARE Signal Pathway

## Abstract

**OBJECTIVES:**

To investigate the molecular mechanism of edaravone (EDA) in improving the post-traumatic brain injury (TBI) dysfunction in learning and memory.

**METHODS:**

*In vitro* and *in vivo* TBI models were established using hydrogen peroxide (H_2_O_2_) treatment for hippocampal nerve stem cells (NSCs) and surgery for rats, followed by EDA treatment. WST 1 measurement, methylthiazol tetrazolium assay, and flow cytometry were performed to determine the activity, proliferation, and apoptosis of NSCs, and malondialdehyde (MDA), lactic dehydrogenase (LDH), and reactive oxygen species (ROS) detection kits were used to analyze the oxides in NSCs.

**RESULTS:**

Following EDA pretreatment, NSCs presented with promising resistance to H_2_O_2_-induced oxidative stress, whereas NSCs manifested significant increases in activity and proliferation and a decrease in apoptosis. Meanwhile, for NSCs, EDA pretreatment reduced the levels of MDA, LDH, and ROS, with a significant upregulation of Nrf2/antioxidant response element (ARE) signaling pathway, whereas for EDA-treated TBI rats, a significant reduction was observed in the trauma area and injury to the hippocampus, with improvement in memory and learning performance and upregulation of Nrf2/ARE signaling pathway.

**CONCLUSIONS:**

EDA, by regulating the activity of Nrf2/ARE signal pathway, can improve the TBI-induced injury to NSCs and learning and memory dysfunction in rats.

## INTRODUCTION

Traumatic brain injury (TBI) has gained increasing attention in clinical research for its contribution to the death and disability of teenagers worldwide (1-3). Dysfunction in learning and memory is a type of brain sequelae in the mechanism of TBI or secondary TBI, which is mainly induced by abnormal activity in the hippocampus. Current evidence has shown that post-TBI dysfunction in learning and memory involves significantly complicated pathologies, including neuronal death and dysfunction in the synapse, hippocampus, or brain network (4-6). So far, decades of efforts have been focused on drugs that are able to improve the dysfunction in learning and memory; however, unfortunately, an effective drug that can be used for the treatment of post-TBI dysfunction in learning and memory is not yet available (7).

TBI, in addition to the injury to the affected region, activates or inhibits a variety of signaling pathways that may further induce the apoptosis or death of nerve cells (8,9), which emphasizes the regulatory role of signal pathways in the treatment of post-TBI dysfunction in learning and memory (8,10). Nrf2, a nuclear transcription factor, can enhance the expression of antioxidant enzymes to antagonize oxidative stress (11,12). TBI usually alters the microenvironment of the brain, specifically the oxygen in the brain (4,13). Thus, Nrf2 activation may be an effective strategy to withstand oxidative stress injury. Accumulating evidence has suggested a key role of Nrf2 in antagonizing oxidative stress injury (12,14). Under oxidative stress, Kelch-like ECH-associated protein 1 (Keap1), an inhibitory protein of Nrf2, regulates the conformal change, which leads to Nrf2 loss (11). Subsequently, Nrf2 is translocated into the nucleus, where it can bind to the antioxidant response element (ARE) to antagonize the oxidative stress induced by reactive oxygen species (ROS) (15).

Edaravone (EDA), a drug that is frequently used for the treatment of TBI and acute ischemic brain injury in clinical practice, has potent lipid solubility and permeability across the blood-brain barrier (16). Additionally, EDA is a highly efficient eliminator of free radicals (16). Hence, it is widely used for the treatment of acute cerebrovascular diseases. Furthermore, in rats, EDA prevents neuronal apoptosis after TBI to ameliorate brain function in rats (17).

To further understand the protective role of EDA in TBI, in this study, we investigated the effect of EDA on the apoptosis of neurons and the expression of Nrf2/ARE signal pathway in the hippocampus. The detailed information is reported as follows:

## MATERIALS AND METHODS

### Animals

Male Sprague Dawley rats weighing between 250-280g were provided by the Model Animal Research Center of Nanjing University. Animal experiments were performed under the supervision of the Ethical Committee for Medical Laboratory Animals of the Affiliated Hospital of Hebei University of Engineering.

### Culture of nerve stem cells (NSCs) of rat hippocampus

NSCs (PR041, Beijing Future Biotech Co., Ltd., Beijing, China) were seeded in DMEM. NSCs were divided into three groups: the hydrogen peroxide (H_2_O_2_) group (NSCs were treated with 30% H_2_O_2_ for 4h), the EDA group (NSCs were treated with 30% H_2_O_2_ and 3 mg/mL EDA for 4h), and the control group (NSCs were treated with normal saline in similar volume).

### Determination of vitality, proliferation, and apoptosis of NSCs

The WST 1 measurement kit (Roche Diagnostics) was used to evaluate the vitality of NSCs (18), whereas the proliferation of NSCs was determined using the methylthiazol tetrazolium (MTT) assay. NSCs in the three groups were seeded in triplicates into a 96-well plate and cultured for 96h, during which cell density was measured every 24h. MTT (5 mg/mL, 20 μL, Beyotime, Shanghai, China) was added to the 96-well plate, which was later transferred into an incubator for incubation (4h, 37°C). The medium was subsequently replaced with 150-μL dimethyl sulfoxide to determine the optical density at 450 nm using a microplate reader (Bio-Rad, Hercules, CA, USA). Apoptosis of NSCs was determined using the Annexin V-FITC/PI Apoptosis Measurement Kit (Sangon Biotech, Shanghai, China).

### Determination of the levels of malondialdehyde (MDA), lactic dehydrogenase, and ROS

NSCs in the three groups were treated at low temperature using ultrasound, followed by the measurement of MDA, LDH, and ROS levels using the corresponding kits provided by Wuhan Zhirong Times Technology Co. Ltd.

### Western blotting and quantitative real-time polymerase chain reaction (qRT-PCR)

Protein samples collected from the NSCs were loaded into sodium dodecyl sulfate-polyacrylamide gel electrophoresis (25 μg/lane), followed by electrophoresis to separate the proteins, which were then transferred onto a polyvinylidene difluoride membrane. Proteins on the membrane were incubated with the corresponding antibodies at 4°C overnight, followed by three washes in phosphate-buffered saline-Tween (PBST), and subsequently probed using secondary antibodies (1:5000; Jackson ImmunoResearch) at room temperature. The resulting immunoblots were further detected using enhanced chemiluminescent reagent and imaged (Amersham Biosciences).

qRT-PCR was performed using the total RNA extracted from the TRIzol reagent from the NSCs and rats, and the expression of targeted genes was quantified using the 2^-ΔΔCt^ method (19).

Sequences:

HO-1 Forward: 5′-TGAAGGAGGCCACCAAGGAGGA-3′

HO-1 Reverse: 5′-AGAGGTCACCCAGGTAGCGGG-3′

SOD1 Forward: 5′-CATCAGCCCTAATCCATCTGA-3′

SOD1 Reverse: 5′-CGCGACTAACAATCAAAGTGA-3′

Nrf2 Forward: CTGAACTCCTGGACGGGACTA

Nrf2 Reverse: CGGTGGGTCTCCGTAAATGG

GAPDH Forward: 5'-CAGTGCCAGCCTCGTCTCAT-3'

GAPDH Reverse: 5'-AGGGGCCATCCACAGTCTTC-3'

### Construction of TBI rat models and plasmids

Anesthesia was induced by intraperitoneal (i.p.) injection of pentobarbital sodium (3 mg/mL) at a dose of 50 mg/kg and sustained by the continuous injection of 2% pentobarbital sodium. Thereafter, a longitudinal incision was made along the middle to expose the part of the skull between the bregma and λ suture lines. The rats were subsequently placed onto the foam under a weight-drop device, where a weight of 450g fell freely through a vertical tube (1.5 m) onto the steel disk. Animals in the sham-operated group underwent the same surgical procedure as those in the TBI group, without a weight-drop impact. TBI rats were randomly divided into four groups: the TBI group (rats were injected with 0.9% normal saline i.p. every 12h for 30 min), EDA group (rats were injected with 3 mg/kg EDA for 30 min every 12h), p-Nrf2 group (rats were injected with the pEGFP-Nrf2 plasmid at a dose of 0.5 mg/kg for 30 min every 12h), and p-NC group (rats were injected with the pEGFP-normal control plasmid at a dose of 0.5 mg/kg for 30 min every 12h), with 10 rats in each group. The following experiments were performed 72h after the TBI. Plasmids were constructed using GenScript (NanjingBiotech Co., Ltd., Nanjing, China).

### Morris water maze

Variants of the Morris water maze paradigm were used to evaluate spatial learning and memory in rats. The time required to determine the hidden platform with a limit of 60s was recorded by a video camera suspended above the maze together with a video tracking system (HVS Imaging, Hampton, UK). The percentage of time spent in the target quadrant and swimming speeds of the animals were investigated after the platform was removed.

### Preparation of brain slices and Nissl staining

On the 5^th^ day after the construction of the TBI models, rats were anesthetized by the injection of 3 mg/mL i.p. pentobarbital sodium to remove the brains. The brains were subsequently fixed in 4% paraformaldehyde for 4h and subsequently placed in 30% sucrose in PBS. In a cryostat (CM1900, Leica, Bensheim, Germany), the brain was incised to prepare 20-μm-thick coronary slices. Nissl staining was performed as previously described. In brief, brain slices were fixed on slides for pretreatment with reagents and covered by a coverslip using neutral resins. Brain slices were subsequently photographed under a microscope (DM5000B, Leica, Bensheim, Germany), and the normal neurons in the CA3 region of the right hippocampus were counted. The final results were represented by the average number of neurons in three rats.

### Data analysis

The Statistical Package for the Social Sciences software version 17.0 was used to perform the data analysis. Differences between two groups or among groups were validated using unpaired Student’s *t-*test or one-way analysis of variance. Statistical significance was set at *p<*0.05. Graphs were prepared using GraphPad Prism version 8.

## RESULTS

### EDA enhances the vitality, proliferation, and apoptosis of NSCs after TBI

**Figure 1:**
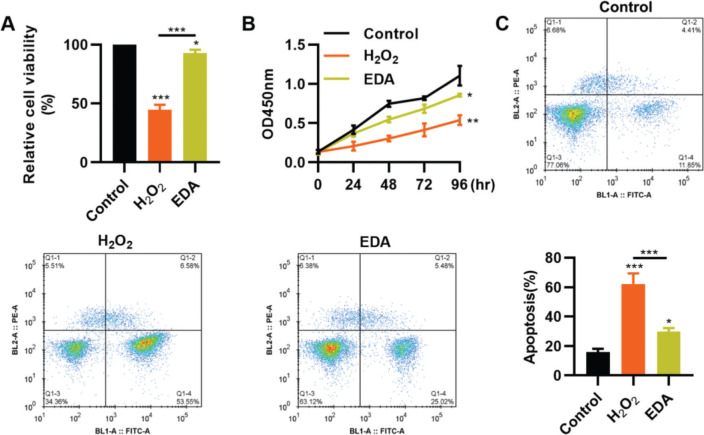
Edaravone (EDA) enhances the vitality and proliferation of hydrogen peroxide (H_2_O_2_)-treated nerve stem cells (NSCs), with a reduction in apoptosis. A, H_2_O_2_ treatment inhibited the vitality of NSCs, whereas EDA treatment rescued the inhibition; B, EDA increased the proliferation of H_2_O_2_-treated NSCs; C, EDA reversed the H_2_O_2_-induced apoptosis of NSCs. *p<0.05, **p<0.01, ***p<0.001. Mann-Whitney U test (A and C), chi-squared test (D).

To clarify the protective role of EDA in the nerve cells of the hippocampus, we simulated the *in vitro* oxidative stress environment of TBI using H_2_O_2_ to treat NSCs. As a result, compared with NSCs in a normal environment, H_2_O_2_ treatment resulted in a sharp decrease in the vitality of NSCs, whereas this change was partially reversed by EDA treatment (Figure 1A, *p<0.05, **p<0.01, ***p<0.001). Similar results were observed in the MTT assay: NSCs in the H_2_O_2_ group had a slower proliferation than those in the EDA group (Figure 1B, *p<0.05, **p<0.01). Flow cytometry results for apoptosis revealed that EDA could improve H_2_O_2_-induced apoptosis in NSCs (Figure 1C, *p<0.05, ***p<0.001).

### EDA relieves the injury of oxidative stress to the NSCs

**Figure 2:**
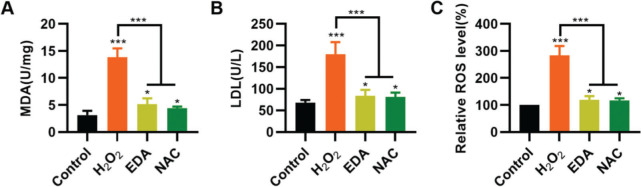
Edaravone (EDA) inhibits the oxidative stress in nerve stem cells (NSCs). A, Malondialdehyde content in the medium of NSCs; B, Lactic dehydrogenase content in the medium of NSCs; C, reactive oxygen species content in the medium of NSCs. *p<0.05, ***p<0.001. Mann-Whitney U test (A-C).

To validate the protective effect of EDA on NSCs from TBI-induced oxidative stress, NSCs were incubated with EDA and H_2_O_2_ for 4h, followed by measurement of MDA, LDH, and ROS levels. Consequently, EDA manifested a potent antioxidant effect, with decreased MDA, LDH, and ROS levels (Figure 2A-C, *p<0.05, ***p<0.001). Additionally, N-acetyl-L-cysteine (NAC), a type of ROS inhibitor, was added to the NSCs, followed by 4h of H_2_O_2_ treatment. As a result, NAC treatment yielded similar results (Figure 2A-C, *p<0.05, ***p<0.001).

### EDA upregulates the Nrf2/ARE signal pathway in NSCs under oxidative stress

**Figure 3:**
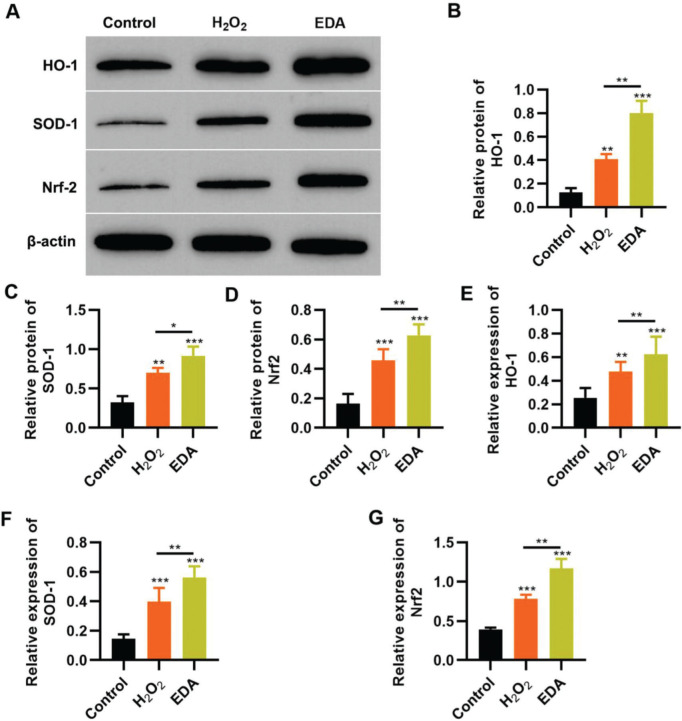
Edaravone (EDA) upregulates the expression of Nrf2/ARE signal pathway. A-C, Western blotting results indicated that EDA upregulated the protein content of heme oxygenase 1 (HO-1) (A), superoxide dismutase-1 (SOD-1) (B), and Nrf2 (C); D-F, Quantitative real-time polymerase chain reaction results indicated that EDA upregulated the mRNA content of HO-1 (D), SOD-1 (E), and Nrf2 (F). *p<0.05, **p<0.01, ***p<0.001. Mann-Whitney U test.

To reflect the status of the antioxidant enzyme system of NSCs in EDA treatment, NSCs were initially incubated with EDA and subsequently with H_2_O_2_ for 4h. Western blot analysis indicated that H_2_O_2_ upregulated the expression of ARE-regulated heme oxygenase 1 (HO-1) and superoxide dismutase-1 (SOD-1), which in turn was further enhanced by EDA treatment (Figure 3A-B, *p<0.05, **p<0.01, ***p<0.001). Moreover, qRT-PCR also yielded results similar to those of western blotting (Figure 3D-E, **p<0.01, ***p<0.001). Additionally, we used western blotting and qRT-PCR to determine the expression of Nrf2 and found that EDA treatment enhanced the protein and mRNA expression of Nrf2 in NSCs (Figure 3C-F, *p<0.05, **p<0.01, ***p<0.001).

### EDA protects the nerves of rats from TBI

**Figure 4:**
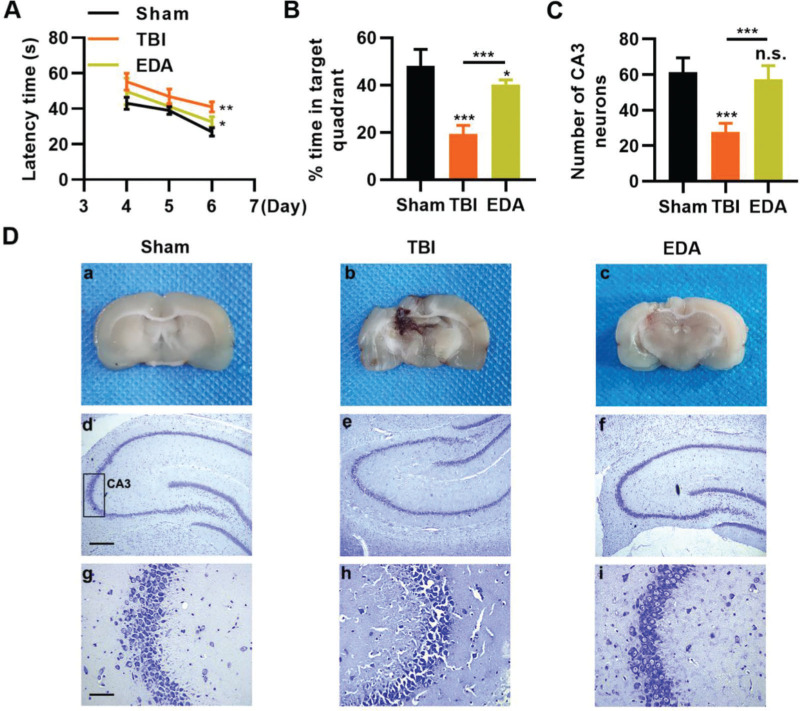
Edaravone (EDA) improves the ability of rats in spatial learning memory. A, Observation of Morris water maze indicated that EDA shortened the escape latency of traumatic brain injury (TBI) rats; B, EDA prolonged the percentage of time of TBI rats spent in the targeted quadrant. *p<0.05, **p<0.01, ***p<0.001. Chi-squared test (A), Mann-Whitney U test (B).

To investigate the protective role of EDA in TBI rats, TBI rats were infused with EDA intravenously (3 mg/kg), followed by the evaluation of learning and memory abilities in rats, and we found that in comparison with the sham group, rats in the TBI group manifested evident dysfunction in spatial learning and memory, with prolongation in escape latency and a decrease in the percentage of time spent in the targeted quadrant (Figure 4A-B, *p<0.05, **p<0.01, ***p<0.001). However, rats in the EDA group showed significant improvements in spatial learning and memory (Figure 4A-B, *p<0.05, **p<0.01, ***p<0.001). Moreover, the increased area of necrosis in the brain, caused by the impact of weight, was also reduced upon EDA injection (Figure 4C-D, **p<0.01). Nissl staining results also revealed that in the CA3 of the hippocampus, TBI rats had 28.3±4.5 neurons, which were significantly lower than in those treated with EDA (60.8±8.3) (Figure 4E-F, **p<0.01).

### EDA upregulates the expression of Nrf2/ARE signal pathway in TBI rats

**Figure 5:**
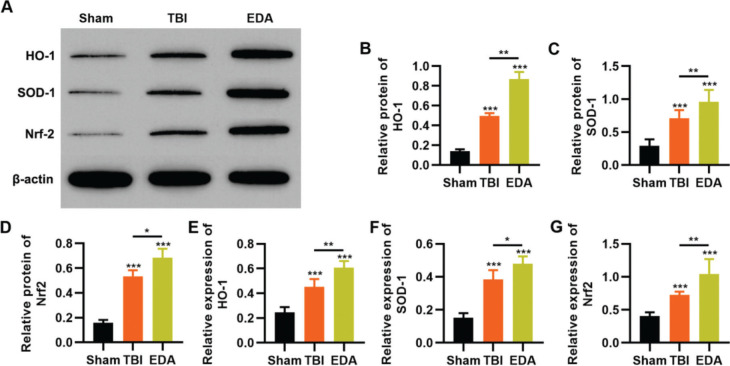
Edaravone (EDA) upregulates the *in vivo* expression of Nrf2/ARE signal pathway in traumatic brain injury (TBI) rats. A-C, Results of Western blotting indicated that EDA could upregulate the protein content of heme oxygenase 1 (HO-1) (A), superoxide dismutase-1 (SOD-1) (B), and Nrf2 (C) in TBI rats; D-F, quantitative real-time polymerase chain reaction results indicated that EDA could upregulate the mRNA content of HO-1 (D), SOD-1 (E), and Nrf2 (F). *p<0.05, **p<0.01, ***p<0.001. Mann-Whitney U test.

To clarify that the protective role of EDA in TBI rats was dependent on the regulation of the Nrf2/ARE signaling pathway, we determined the effect of EDA treatment on the expression pattern of the Nrf2/ARE signaling pathway by western blotting and RT-PCR. TBI rats showed upregulation of HO-1, SOD-1, and Nrf2, which were further upregulated in the EDA-treated TBI rats (Figure 5 A-F, *p<0.05, **p<0.01, ***p<0.001).

### p-Nrf2 plasmid improves the function of TBI rats in learning and memory

**Figure 6:**
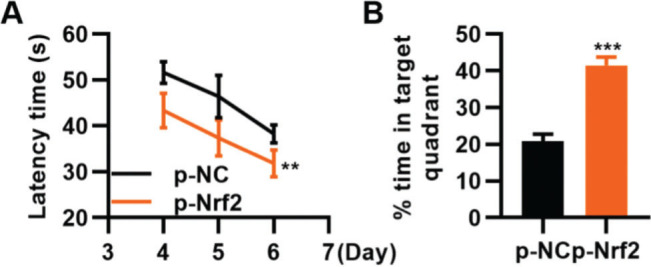
Transfection of p-Nrf2 plasmid enhances the ability of traumatic brain injury (TBI) rats in spatial learning and memory. A, Results of the Morris water maze indicated that p-Nrf2 transfection shortened the escape latency of TBI rats; B, Rats in the p-Nrf2 group had an increased percentage of time spent in the targeted quadrant. **p<0.01, ***p<0.001. Chi-squared test (A), Mann-Whitney U test (B).

To confirm that the activation of the Nrf2/ARE signaling pathway can improve TBI, we transfected TBI rats with p-Nrf2 plasmid and found that p-Nrf2 transfection improved the function of TBI rats in spatial learning and memory compared to TBI rats transfected with p-NC plasmid (Figure 6A-B, **p<0.01, ***p<0.001).

## DISCUSSION

As a common drug frequently used in clinical practice, EDA shows promising efficacy in the apoptosis of nerve cells caused by ischemic injury in the brain (17,20). Ischemic injury is mainly induced by free radicals in ischemic tissues, generated by oxidative reactions (17,21). TBI also results in massive oxidative stress in the affected tissues, which may further influence the nerves (22). Hippocampal injury-induced dysfunction in learning and memory is one of the consequences of TBI (23-25), which is mainly attributed to oxidative stress-induced injury to the NSCs (26).

EDA, as a typical eliminator of free radicals, can enter cells to inhibit the release of free radicals (17). EDA can suppress the free radical-induced degeneration of neurons and the death of surrounding cells (27), and some researchers have also found that EDA could also prevent TBI-induced cognitive impairment by inhibiting oxidative stress and alleviating axonal injury (28). Hence, EDA is believed to be a neuroprotective compound (28). Recently, EDA has been considered as an available candidate for the treatment of oxidative stress-induced neurodegenerative diseases (29). Nevertheless, studies investigating the role and potential mechanism of EDA in the treatment of TBI are insufficient. Therefore, in this study, we investigated the antioxidative role of EDA in the oxidative stress models of NSCs and the regulatory mechanism in improving TBI-induced injury to the hippocampus of rats.

In this study, we performed *in vitro* experiments on NSCs and *in vivo* experiments on TBI rats and found that EDA could improve the 30% H_2_O_2_-induced apoptosis of NSCs, with a significant enhancement of the vitality and proliferation of NSCs, suggesting that EDA could improve H_2_O_2_-induced oxidative stress and protect hippocampal NSCs by activating the Nrf2/ARE signaling pathway. As such, we inferred that EDA plays a protective role in the rat hippocampus. Thus, we constructed TBI models in rats and observed that EDA injection can reduce the injury volume of TBI and protect hippocampal neurons from TBI. Moreover, the dysfunction in spatial learning and memory that was observed in TBI rats was also reversed by EDA injection, which suggested that EDA could improve the status of the hippocampus of TBI rats to ameliorate the function of TBI rats in learning and memory. Findings derived from the study of the molecular mechanism revealed that the protective mechanism of EDA for neurons may depend on the activation of the Nrf2/ARE signaling pathway, similar to the results of *in vitro* experiments.

The Nrf2/ARE signaling pathway involves Nrf2, a transcription factor, and ARE, its cis-acting element (15). In cells facing intensified internal or external oxidative stress, Nrf2 is released by the ubiquitination of Keap1, an inhibitor of Nrf2 (14). Activated Nrf2 is translocated into the nucleus to bind to ARE, through which it can activate the expression of downstream antioxidative genes (15). Accumulating evidence has also indicated that the activation of the Nrf2/ARE signaling pathway can increase antioxidative ability and reduce oxidative stress-induced injury to cells (11,30-32).

In summary, the findings of this study indicate that EDA could abate TBI-induced oxidative stress in hippocampal neurons *in vivo* and *in vitro*. This depends on the activation of the Nrf2/ARE signaling pathway by EDA, which could further decrease the intracellular levels of MDA, LDH, and ROS; mitigate hippocampal damage; and improve the function of TBI rats in spatial learning and memory.

## AUTHOR CONTRIBUTIONS

Li X and Yu J designed the study. Yu J and Ma D performed the study. Weng X analyzed the data. Li X and Yu J wrote the manuscript. All authors contributed to the editorial changes in the manuscript. All authors have read and approved the final version of the manuscript.
